# Fairness in classroom assessment: development and validation of a questionnaire

**DOI:** 10.1186/s40468-022-00162-9

**Published:** 2022-06-01

**Authors:** Afsheen Rezai

**Affiliations:** English Language Department, Faculty of Literature and Humanities, Ayatollah Burojerdi University, Burojerd City, Lorestan Province 68571-14597 Iran

**Keywords:** Fairness, Classroom assessment, Confirmatory factor analysis, Exploratory factor analysis

## Abstract

Although fairness in assessment practices has gained noticeable attention over the recent years, there has been a long-lasting study to design and validate a questionnaire to measure it from a psychometric perspective. Thus, this study aims to develop and validate a questionnaire with adequate psychometric properties to measure fairness in classroom assessment. Using a random sampling method, two samples of male and female university students for the first pilot (*n* = 128) and the second pilot (*n* = 360) were selected from Ayatollah Borujerdi University and Lorestan University. Drawing on the past literature, a pool of items (*n* = 118) were extracted and subjected to a 12-step systematic procedure, including content analysis and sampling; creating an item bank; running the first pilot; creating item pool one; expert judgment to evaluate the sub-scales; running an interview and think-aloud protocol; running Cronbach’s alpha; running the second pilot; running exploratory factor analysis, confirmatory factor analysis, and Cronbach’s alpha; creating item pool two; expert review; and translation and translation quality check. Findings yielded a 110-item questionnaire with 10 sub-scales: learning materials and practices (18 items); test design (24 items); opportunities to demonstrate learning (8 items); test administration (21 items); grading (11 items); offering feedback (6 items); tests results interpretation (5 items); decisions based on tests results (3 items); test results consequences (4 items); and students’ fairness-related beliefs and attitudes (10 items). The hope is that this questionnaire can serve research and educational purposes.

## Introduction

The term ‘fairness’ is defined as “the quality of treating people equally or in a way that is right or reasonable” by the Cambridge Advanced Learners’ Dictionary. Similarly, it is defined as “marked by impartiality and honesty: free from self-interest, prejudice, or favoritism” by the Merriam-Webster Dictionary. These definitions infer that assessment practices (APs) are fair if they are ‘free of favoritism’ and ‘free of biases’. According to Kane (2010), APs are considered as fair if they do not unduly privilege a particular group of test-takers. Cole and Moss (1989) note that unfairness in APs is perceived the “differential validity of a given test score for any definable, relevant subgroup of test-takers” (p. 205). In simple terms, fairness is the “absence of bias, equitable treatment of all test-takers in the testing process, and equity in opportunity to learn the material in an achievement test” (Educational Testing Service, 2014, p. 57).

Of particular note is that the notion of ‘fairness’ differs from the notion of ‘justice’. According to McNamara and Ryan (2011), fairness refers to APs’ technical (psychometric) qualities. They define fairness as “the extent to which the test quality, especially its psychometric quality, ensures procedural equality for individual and subgroups of test-takers and the adequacy of the representation of the construct in test materials and procedures” (p. 163). In contrast, justice deals with “the social consequences of test use along with the value implications and the sociopolitical ideologies underlying the test constructs” (Karami, 2013). In simple terms, fairness pertains to the use of test results and their interpretation. However, justice relates to the social consequences that test use and interpretation create for test-takers.

To conceptualize the term of fair assessment, we need to refer to the binomial equality-equity (Baniasadi et al., 2022; Murillo & Hidalgo, 2020; Nisbet, 2019; Tierney, 2014, 2016). According to Nisbet and Shaw (2019), fair assessment can be linked with either the notion of ‘equality’ or the notion of ‘equity’. The notion of equality aims to ensure that assessment conditions, such as learning materials, resources, time, and place are equal for all test-takers. Equal assessment, in other words, calls for “the same administration, content, scoring, and interpretation of results” (Murillo & Hidalgo, 2020, p. 2) to achieve objectivity. On the other hand, as the educational opportunities are not available for all test-takers to the same degree, equality is not enough to ensure fair assessment (Hamid et al., 2019; Suskie, 2002; Shiba et al., 2015; Scott et al., 2014; Tierney, 2016). Therefore, fair assessment requires equity. Equity means that APs should be adapted to test-takers’ needs and characteristics (Murillo & Hidalgo, 2020; Tierney, 2016). As Murillo and Hidalgo (2020) note, equity is met in APs by administering “multiple assessments with different instruments to make sure that student diversity is properly taken into account throughout the assessment” (p. 2). Considering the equality-equity binomial, APs are fair when it does minimize the bias against all test-takers (Bazvand & Rasooli, 2022; Zieky, 2016).

Crucially for the current study, there has been a long-lasting paucity of a reliable, well-validated questionnaire to measure fairness in classroom assessment (CA). It is essential to design and validate a questionnaire that can measure the fairness of assessment practices in different contexts in a psychometric way. To fill in this gap, the present study purported to develop a reliable, well-validated questionnaire to measure fairness in CA from Iranian university students’ perceptions. It is hoped that the results of this study can be useful for different testing stakeholders to check if the assessment practices administered in different contexts meet the requirements in terms of fairness.

## Literature review

One of the key concepts that has constantly been considered as an integral part of quality APs is fairness (Baniasadi et al., 2022; DeLuca, 2012; DeLuca et al., 2016; Green et al., 2007; Kunnan, 2000, 2004, 2018; Rezai et al., 2021; Tierney, 2014; Xu & Brown, 2016). The previous studies’ findings have disclosed that fair APs are closely correlated with students’ motivation for learning (Chory-Assad, 2002), students’ engagement in learning (Berti et al., 2010), and students’ level of academic achievement (Holmgren & Bolkan, 2014). Based on the previous studies’ findings, fair APs have positive effects on students’ self-efficacy (Vallade et al., 2014), political trust (Abdelzadeh et al., 2015), teachers’ satisfaction (Wendorf & Alexander, 2005), school authority and teachers’ legitimation (Nelson et al., 2014), and students’ evaluation of their teacher expertise (Gotlieb, 2009). However, taking a glance at the available literature reveals that there has been a lack of consensus on what makes APs fair (Green et al., 2007; Liu et al., 2016; Rasooli et al., 2018).

Different scholars have tried to illuminate the basic features of fair APs. For example, Peters et al. (2017) consider APs as fair if (a) they are not used as a mechanism for classification but as a diagnostic tool, (b) they are used to improve student learning not as an external tool to measure students’ performance, and (c) they are used to even out the overall students’ evaluation, not as a punishment tool for students who do not meet the intended requirements. Additionally, Pettifor and Saklofske (2012) note that one of the best ways to transfer educational APs to fair practices is by making test-takers familiar with evaluation criteria. They add that those evaluation criteria should be co-defined jointly by test-makers and test-takers. Moreover, Stobart (2005) maintains that to achieve fairness in APs, test-makers should make sure that there is no bias against test-takers regarding their gender, ethnicity, nationality, and socioeconomic status. Likewise, for Kyaruzi et al. (2018), APs are fair when they are tailored to test-takers’ needs and characteristics.

In the past literature, a range of studies has been conducted to verify the fundamental features of fair APs from teachers’ and students’ perspectives. In an early attempt, Green et al. (2007) examined teachers’ perceptions about fair challenges in summative tests. Their findings documented that confidentiality, communication about grading, and multiple assessment opportunities received the highest value from the participants’ perspectives. Moreover, in another study, Tierney et al. (2011) examined how Canadian teachers assessed their students. Their findings evidenced that (a) teachers should take into account their students’ progress during the course; (b) skills related to products than procedures should be given attention; (c) professional judgment along with standards-based grades should be used by teachers to assess their students’ learning; and (d) teachers should provide students with enough feedback about their performance and grades. Additionally, in research by Segers and Tillema (2011), teachers’ conceptions about fair APs in the Netherlands were investigated. Their results documented that APs are considered as fair if they met some criteria: being useful for student learning, being beneficial to demonstrate what students have learned, being interesting for students, being helpful to create a collaborative climate in the classroom, and serving to exert accountability. Furthermore, Tierney (2014) carried out a multi-case study to re-conceptualize fair assessment from the Canadian primary and secondary teachers’ perspectives. His results documented that the participants perceived APs as fair if they equitable for all test-takers, offer multiple learning opportunities to all test-takers, transparent, create a trustful environment in the classroom promoting critical reflection, and avoid an equal assessment for all test-takers. Likewise, Scott et al. (2014) did mixed-methods research to disclose the Canadian teachers’ perceptions about fairness in CA. Their findings revealed that fair assessment is more connected to the notion of equity. Moreover, their results showed that APs are perceived as fair if they meet five criteria: (a) test makers have a clear understanding of the effects of tests on test-takers and their families; (b) tests are designed and administered based on test-takers’ needs and characteristics (e.g., ability level, gender, socioeconomic status, culture, and language); (c) all testing stakeholders have the right to express their voices and concerns about assessment malpractices; (d) test-takers and their families are not overwhelmed by the frequency, intensity, and intrusion of APs; and (e) APs are not used as instruments to punish or reward test-takers. Finally, Murillo and Hidalgo (2020) conducted a phenomenographic study to disclose fairness in APs from teachers’ conceptions in Spain. Their findings indicated that the participants’ conceptions of fair assessment were closely related to the principle of equality and equity. Additionally, their findings unveiled that the participants’ perceptions of fair assessment were influenced by the school context.

University students’ perceptions about fair assessment were examined at Southwestern University by Pepper and Pathak (2008). Their findings indicated that the participants perceived APs as fair if there was explicitness in assessment administration and grading criteria, frequent feedback, and proactivity in the assessment process. Further, in research by Murillo and Hidalgo (2017), primary and secondary school students’ perceptions about fair APs were explored in Spain. They found that, on the one hand, the participants’ perceptions about fair assessment were associated with equality, objectivity, transparency, and evaluation of class content. On the other hand, the participants’ perceptions about fair assessment were related to equity which included some ideas, such as diversification of tests, adaptation, and qualitative assessment. Likewise, in another study, Wallace (2018) explored Taiwanese university L2 learners’ (*n* = 83) perceptions about the fairness of a single test administration. The participants reported that the test administration had a high level of interactional fairness and a high level of procedural fairness. However, the level of distributive fairness was moderate. These findings mean that for the participants, interactions with their teachers were respectful and testing procedures were followed equally for all test-takers. Still, the test scores did not represent their performance adequately. Moreover, Rasooli et al. (2019) tried to conceptualize fair assessment from Iranian university teachers’ perceptions. Their findings evidenced that the participants’ perceptions of assessment fairness included three principles: distributive justice, procedural justice, and interactional justice. In addition, their participants perceived the procedures for outcome distributions, the communication procedures, and the interpersonal relationships as crucial in the conceptualization of assessment fairness. Likewise, in a systematic meta-ethnographic study, Rasooli et al. (2018) tried to present a comprehensive conceptualization of assessment fairness in the classroom with a dominant focus on how fair APs affect student learning. They found that APs are perceived as fair if (a) students have enough opportunities for learning and enough opportunities for demonstrating learning; (b) there is transparency, consistency, and justification in APs; (c) there are suitable accommodations; (d) APs follow the ‘do no harm principle’ and classroom environment is constructive; (e) there is no score pollution; and (d) students have opportunities to do group work and assess their peers’ performance. Finally, Bazvand and Rasooli (2022) explored Iranian postgraduate university students’ perceptions of fairness in classroom assessment within the higher education context. They found that the participants’ perceptions of fairness had been affected by ‘equity principle’ and ‘interactional fairness principle’.

### Fair assessment models

In the past literature, some models have been presented to conceptualize fair assessment. Here, we review critically three influential ones. One of the first comprehensive models to illuminate the concept of fairness was presented by Kunnan (2004). This model consists of five features: *validity, absence of bias, access, administration*, and *social consequences*. The feature of validity means that the required evidence of ‘content representativeness or coverage evidence’, ‘construct or theory-based validity evidence’, ‘criterion-related validity evidence’, and ‘reliability’ is collected. Content representativeness or coverage evidence means that testing practices represent test domain adequately. Construct or theory-based validity evidence suggests that testing practices represent the test domain adequately. Criterion-related validity evidence means that “the test scores under consideration meet criterion variables such as school or college grades and on the job-ratings, or some other relevant variable” (Kunnan, 2004, p. 37). Reliability indicates that test results are consistent in terms of stability (e.g., test scores’ consistency on different testing occasions), alternative form evidence (e.g., test scores’ consistency between two or more different forms of a test), inter-rater evidence (e.g., test scores’ consistency between two or more raters), and internal consistency evidence (e.g., “in the way test items measure a construct function” (Kunnan, 2004, p. 37)*.* The feature of the absence of bias means that the required evidence of ‘offensive content or language’, ‘unfair penalization based on test taker’s background’, and ‘disparate impact and standard setting’ is gathered. Offensive content or language means that the content of tests is not offensive for test-takers with different backgrounds (e.g., gender, religion, age, first language and culture, and nationality). Unfair penalization based on test-taker’s background means that the content of tests does not cause unfair penalization due to the membership of a test-taker to a particular group or community. Disparate impact and standard setting suggest that there is no bias against a group of test-takers in terms of different performance and outcomes. The feature of access means that the needed evidence for ‘educational access’, ‘financial access’, ‘geographical access’, ‘personal access’, and ‘conditions or equipment access’ is collected. The educational access means that all test-takers have equal opportunities to learn the content and they have equal opportunities to become familiar with testing practices. The financial access means that all test-takers afford to pay for tests’ expenses. The geographical access means that all test-takers have easy access to test sites. Personal access means that test accommodations are appropriate for all test-takers even those with physical and learning disabilities. Conditions or equipment access means that “takers are familiar with the test-taking equipment (such as computers), procedures (such as reading a map), and conditions (such as using planning time)” (Kunnan, 2004, p. 38). The administration feature implies that the required evidence of ‘*physical conditions*’ and ‘*uniformity or consistency*’ is gathered. The physical conditions suggest that test administration conditions and facilities (e.g., light, temperature level, chair) are appropriate. The consistency means that test administration conditions are consistent across tests sites. However, the uniformity infers that all test-takers take tests under the same conditions. The social consequences feature implies that the required evidence of ‘washback’ and ‘remedies’ is collected. The washback treats test effects on instructional practices (e.g., educational materials, ways of teaching, ways of learning, and test-taking strategies). The remedies refer to “remedies offered to test takers to reverse the detrimental consequences of a test, such as re-scoring and re-evaluation of test responses, and legal remedies for high-stakes tests (Kunnan, 2004, p. 39).

The second model is the Assessment Use Argument (AUA), presented by Bachman and Palmer (2010). AUA includes four claims, namely assessment records, interpretations, decisions, and consequences. For each claim, they offer one or more assumptions requiring theoretical and empirical support to establish a compelling validity argument. They argue if interpretations are meaningful, impartial, generalizable, relevant, and sufficient; decisions are values sensitive and equitable; consequences are beneficial; and assessment records are consistent. Under AUA, test results interpretation and use are valid if they are stated clearly and are supported by strong evidence. In simple terms, to evaluate the validity of test results interpretations and uses, there is a need for the completeness and coherence of a network of inferences and assumptions (or an argument) (Kane & Burns, 2013). It should be stressed that the researcher used the above-alluded studies and models to extract the sub-scales and items.

As this review demonstrates, while the above-alluded studies and models have been noticeable attempts to present a comprehensive definition of fair assessment construct, none of them have purported to develop and validate a psychometrically sound questionnaire to measure fairness in CA. Therefore, the present study is the first attempt to develop and validate a questionnaire with sound psychometric properties to gauge fairness in CA.

## Method

As pointed out above, this study aims to develop and validate a questionnaire to measure fairness in CA. The researcher went through a systematic, 12-step design, and validation procedure for the development of an assessment fairness questionnaire (AFQ). The primary purpose was to produce a psychometrically sound questionnaire by ensuring that the reliability and validity criteria were met well. This systematic procedure was based on the practices recommended by leading scholars in social sciences (Artino Jr et al., 2014; Dörnyei, 2003) and followed by Salehi and Jafari (2015). Table 1 presents the steps taken to design and validate AFQ. Each of the steps is detailed below.

**Table 1:** Twelve-step questionnaire development and validation procedures

Step 1	Content analysis and sampling
Step 2	Creating an item bank
Step 3	Running the first pilot
Step 4	Creating item pool one
Step 5	Running expert judgment to evaluate the sub-scales
Step 6	Running an interview and think-aloud protocol
Step 7	Running Cronbach’s alpha
Step 8	Running the second pilot
Step 9	Running exploratory factor analysis, confirmatory factor analysis, and Cronbach’s alpha
Step 10	Creating item pool two
Step 11	Running expert reviews
Step 12	Running translation and translation quality check

The first step was content analysis and sampling. In line with Clément et al. (1994), the past literature, including definitions, models, and instruments was meticulously inspected to extract and verify the most frequent and relevant components of assessment fairness. The analysis yielded 10 overarching sub-scales: (1) learning materials and practices; (2) test design; (3) opportunities to demonstrate learning; (4) test administration; (5) grading; (6) offering feedback; (7) tests results interpretation; (8) decisions based on tests results; (9) test results consequences; and (10) students’ fairness-related beliefs and attitudes. As teaching and testing are complementary, the emerged sub-scales included both teaching and testing processes. The teaching sub-scale covered opportunities that test-takers may have to learn educational materials. The testing sub-scales comprised the steps that should be taken to implement quality APs in the classroom.

The second step was creating an item bank. To meet the current study’s goals, over 203 items were collected to make an item bank. For this purpose, the researcher went through the available literature meticulously to extract and verify the items related to the sub-scales of assessment fairness. The items were designed based on three dimensions of fairness: *distributive justice*, *procedural justice*, and *interactional justice* (Green et al., 2007; Greenberg, 1993; Kunnan, 2000, 2004; Rasooli et al., 2018, 2019; Tierney, 2014; Wallace, 2018). Distributive justice examines if outcomes are distributed based on three principles: ‘*equity*’, ‘*equality*’, and ‘*need*’. The principle of *equity* examines if the comparison of the ratio of outcome distribution of a test-taker is same with that of a similar test-taker. The principle of *equality* inspects if the outcomes are equally distributed among test-takers. The principle of *need* probes if the outcomes are distributed based on test-takers’ needs. Procedural justice treats the fairness of the procedures for outcome distributions based on five principles: *consistency*, *bias suppression*, *accuracy*, *correctability*, *voice*, and *ethicality*. The principle of consistency checks out if the procedures are implemented consistently. The principle of bias suppression investigates if the procedures are implemented neutrally. The principle of accuracy surveys if the procedures are implemented in accurate ways. The principle of correctability examines if procedures get corrected if they are identified as implemented wrongly. The principle of ethicality scrutinizes if the procedures meet the ethical considerations. Interactional justice refers to the fairness of interactions and communications among testing stakeholders based on four principles: *respect*, *property*, *truthfulness*, and *justification*. The principle of respect examines if relationships among testing stakeholders are respectful. The principle of property inspects if the communication of information among testing stakeholders is respectful. The principle of truthfulness probes if the communication of information among testing stakeholders is honest. The principle of justification checks out if the explanation of outcomes and procedures is logical. In order to develop quality items, the researcher constantly reviewed and contrasted the items of the item bank with AFQ. It should be noted that the researcher took no item of the item pool directly (constructed in the succeeding stages) from this item bank. The major role of this item bank was to increase the quality of the items in the item pools.

The third step was creating the item pool one. The researcher developed the first version of the items. Due to the additive nature of Likert scale self-analysis questionnaires, the number of items assigned to each sub-scale is of paramount importance. The reason for this is reliability where the number of items for each sub-scale should not be less than four items (Dörnyei, 2003). The researcher wrote the items in Persian and English concurrently. Considering the items in the item pool one, the researcher wrote the first draft of AFQ with 118 items. It should be noted that the researcher constantly read, edited, and revised the draft. Next, the items written in natural English were simplified lexically. As the intended respondents were supposed to be students with different English proficiency levels, simplifying the items would increase the readability of AFQ for a large scale of respondents. In turn, as Radhakrishna (2007) stresses, this led to the increase of AFQ reliability and validity. It should be highlighted that the researcher used the Corpus of Contemporary American English (COCA) constantly to examine the simplicity of the words of the item pool. In particular, the researcher chose the words that were among the first 2000 words of the corpus in terms of frequency.

The fourth step was running expert judgment to evaluate the sub-scales. The researcher used expert judgment to increase the content validity of AFQ and make sure if the sub-scales emerged from the analysis of the literature could be included in the item pool. Using a sub-scale evaluation checklist, the researcher invited four university professors specialized in applied linguistics at Lorestan University to judge if the sub-scales were necessary for AFQ. It should be noted that expert judgment was used as a pre-testing method before running the psychometric validity procedures. As Olson (2010) notes, expert judgment can be used to “discern questions that manifest data quality problems” (p. 295). The sub-scales evaluation checklist consisted of 10 items. For example, the first item asked, “Do you agree that providing opportunities to demonstrate learning is an essential component for assessment fairness in the classroom? If yes, how much? The Likert type used for the evaluation of sub-scales checklist was a three-point Likert-scale. It included three items, namely not essential, somehow essential, and very essential. Then, the experts were asked to provide their reasons for their choice. The experts’ judgment revealed that all the sub-scales were needed, and therefore, no sub-scale was deleted.

The fifth step was running an interview and think-aloud protocol. The researcher used a verbal report protocol to interview 15 students from the target samples. The reason was to identify the vague items and make sure the response validity of the items. The participants were invited to report on the items lacking the required readability. During the online interviews running on Adobe connect platform, the participants carefully went over the items one by one, thought aloud, and expressed their views and feelings. The researcher asked if the items were clear enough to the participants. If the participants expressed a problem with the items, they were stopped and asked for the defective aspects of the items. It is worthy to note that the Persian version of the items was used and the interviews were run in Persian so that the interviewees can express their ideas and conceptions with ease.

The sixth step was running the first pilot. To pilot AFQ for the first time, a total of 128 university students were selected using a random sampling method at Ayatollah Borujerdi University and Lorestan University, Iran. The underlying reason for selecting the participants was their easy availability and their great deal of experience with test-taking. They included males (*n* = 82) and females (*n* = 38) and their ages ranged from 19 to 45. They were B.A. (*n* = 105) and M.A. (*n* = 23) undergraduate students who majored in English literature, applied linguistics, and linguistics. To access the participants, the first researcher referred to the Deputy of Education of Ayatollah Borujerdi University and Lorestan University and explained the present study’s objectives. Both Deputies of Education allowed the researcher to meet the department heads of English language and literature. Having described in detail the present study’s objectives, the department heads permitted the researcher and colleagues to run the study with the cooperation of their students. Since the present study was conducted during the outbreak of the COVID-19, the students were not present on the campus and, therefore, the researcher could not meet them in person. The researcher got the students’ phone numbers and sent a podcast voice to them via WhatsApp. During the COVID-19 pandemic, all university students installed WhatsApp on their phones to be in touch with their university teachers, university officials, and classmates. The podcast voice explained the current study’s objectives and asked if they agree to fill in the questionnaire. A total of 128 students agreed willingly to fill in the questionnaire. Then, the researcher sent a digital format of AFQ to them. It should be noted that AFQ started with digital written consent (in Persian) and if the participants agreed with its content, they could move on to the next stage to fill in the questionnaire. During answering the items, the participants could contact the researcher to raise their problems with the items.

The seventh step was running Cronbach’s alpha. In line with Dörnyei (2003), in the pilot phase, the internal consistency indexes were used to reduce the problematic items of the item pool one. For this purpose, the researcher used Cronbach’s alpha to delete the problematic items. Based on the results, the items whose Cronbach’s alpha was less than 0.70 (8 items) were deleted. In total, the Cronbach’s alpha of 110 items was larger than 0.80.

The eighth step was running the second pilot. According to the results of the first expert judgments and Cronbach’s alpha, 10 sub-scales with 110 items were verified. Hence, the second item pool included 110 items. In this step, AFQ was distributed among 360 university students selected the through random sampling method. They included male (*n* = 245) and female (*n* = 115) students and their ages ranged from 19 to 47. They were B.A. (*n* = 270) and M.A. (*n* = 90) undergraduate students majoring in English literature, applied linguistics, and linguistics. The procedures explained in the sixth step were followed to achieve the participants in the second pilot two. It should be noted that the participants were ensured that their responses would remain confidential and they would be kept informed about the final findings.

The ninth step was running exploratory factor analysis (EFA), confirmatory factor analysis (CFA), and Cronbach’s alpha. According to Riazi (2016), EFA is a statistical test used to disclose the underlying theoretical foundations of a topic by reducing data to a smaller set of variables. However, CFA is a statistical test used to verify the factor structure of a set of observable variables. The researcher subjected the 110 items of AFQ to an EFA to explore its factorial structure. Afterward, the researcher run a CFA to check if the factors emerged in the EFA were confirmed. Next, the researcher examined the internal consistency of items using Cronbach’s alpha. The primary purpose was to identify and delete the defective items creating factor pollution and alpha reduction from item pool two.

The tenth step was creating the item pool two. To make it, the researcher made some modifications. He rewrote and replaced the items deleted in the statistical analysis section above with new items. In line with the current study’s aims, the researcher simplified some items more in terms of grammar and lexicon to improve their readability. He chose simple tenses, changed the sentences’ voices at times, and reversed and paraphrased some items to become simpler.

The eleventh step was running expert reviews. Two associate professors in Applied Linguistics at Tehran University were invited to review and comment on the items. The researcher referred to their offices and asked them kindly to examine the items based on six criteria: double-barreled, vague, unrepresentative, hard, sensitive, and burdensome (Dörnyei, 2003). In light of the professors’ comments, some minor modifications were made in regard to the language of the items. However, no item was deleted from the final version of AFQ.

The last step was running translation and translation quality check. As pointed out above, the researcher wrote the items in English and Persian from the beginning and changed both equivalents of the items concurrently. The reason for this was making parallel versions of AFQ. As mentioned above, in the first and second pilot phases, the Persian version of the items was used. There were some reasons for this. First, the participants had different language proficiency levels. Second, misunderstanding of the items may have jeopardized the reliability and validity of the participants’ responses. Third, responding to the questionnaire in Persian was naturally less anxiety-provoking for the participants. Fourth, answering the questionnaire in Persian was less time-consuming for the participants. It should be stressed that the researcher checked the quality of the translation in two ways. In the first way, they invited 15 students to report on the clarity of the translations and highlight the vague words, phrases, and sentences. In the second way, the researcher invited two experts in translation to check the clarity of the translations and the equivalence between the Persian and English items. Based on their comments, some modifications were made to the defective translations.

## Results and discussion

To achieve the intended aims, the researcher used Cronbach’s alpha, EFA, and CFA. The researcher used EFA and CFA in the second pilot. He used SPSS version 22 to run EFA analyses and he used the analysis of moment structures (AMOS) V. 21 program to run CFA. The reason for using AMOS was that it supports SEM (structural equation modeling) and has a graphical interface and is diagram-based (Kline, 1998). The results of internal consistency reliability for the pilot one was 0.72 (Table 2).

**Table 2:** Reliability of the first version of the questionnaire

	Number of Items	Cronbach’s alpha
Overall	118	0.726

The researcher subjected the 110 items of the questionnaire to EFA with oblique rotation (direct oblimin) to explore the factorial structure of AFQ in the sample. The sampling adequacy for the analysis, KMO = 0.92 (‘marvelous’ according to Kaiser & Rice, 1974) was verified by the Kaiser-Meyer-Olkin measure. Bartlett’s test of sphericity was *χ*^2^ (5995) = 39803.55, *p* < .05, indicating that the correlation structure is adequate for factor analyses. An initial analysis was run to obtain eigenvalues for each factor in the data. Ten factors had eigenvalues over Kaiser’s criterion of 1 and in combination explained 73.26 % of the variance. We retained 10 factors because of the large sample size and the convergence of the screen plot and Kaiser’s criterion on this value. Table (rotated component matrix) shows the factor loadings after rotation. The table shows the results of the final version of TF. As Field (2013) asserts, since no item had loadings below 0.4, none of the items were deleted from the final version. The items clustering on the same factor suggest that factor 1 represents ‘test design’, factor 2 represents ‘test administration’, factor 3 represents ‘learning materials and practices’, factor 4 represents ‘grading’, factor 5 represents ‘students’ fairness related beliefs and attitudes’, factor 6 represents ‘opportunities to demonstrate learning’, factor 7 represents ‘offering feedback’, factor 8 represents ‘tests results interpretation’, factor 9 represents ‘tests results consequences’, and factor 10 represents ‘decisions based on tests results’ (Table 3).

**Table 3:** Factor loadings for the TF (*n* = 360, 101 items) or rotated component matrix

Items	Component									
	1	2	3	4	5	6	7	8	9	10
Q1			.832							
Q2			.821							
Q3			.857							
Q4	.837									
Q5	.851									
Q6	.835									
Q7	.845									
Q8	.845									
Q9	.832									
Q10						.844				
Q11						.855				
Q12						.844				
Q13		.847								
Q14		.834								
Q15		.830								
Q16		.835								
Q17		.830								
Q18		.860								
Q19		.839								
Q20				.836						
Q21				.839						
Q22				.863						
Q23				.851						
Q24				.828						
Q25				.831						
Q26							.865			
Q27							.852			
Q28							.842			
Q29							.854			
Q30								.872		
Q31								.871		
Q32								.861		
Q33								.859		
Q34										.894
Q35										.857
Q36									.868	
Q37									.879	
Q38									.928	
Q39					.854					
Q40					.849					
Q41					.848					
Q42					.828					
Q43					.873					
Q44	.846									
Q45	.825									
Q46	.835									
Q47	.850									
Q48	.839									
Q49			.822							
Q50			.836							
Q51			.843							
Q52			.848							
Q53			.837							
Q54			.818							
Q55			.831							
Q56	.846									
Q57	.843									
Q58						.814				
Q59						.858				
Q60						.835				
Q61		.829								
Q62		.845								
Q63		.832								
Q64						.837				
Q65		.836								
Q66		.855								
Q67		.844								
Q68		.846								
Q69		.839								
Q70				.839						
Q71				.858						
Q72							.871			
Q73										.881
Q74									.892	
Q75					.846					
Q76					.922					
Q77					.852					
Q78					.844					
Q79					.843					
Q80								.869		
Q81							.846			
Q82				.844						
Q83				.834						
Q84		.844								
Q85		.853								
Q86			.829							
Q87			.816							
Q88			.823							
Q89			.837							
Q90			.819							
Q91			.822							
Q92			.817							
Q93			.836							
Q94	.845									
Q95	.853									
Q96	.841									
Q97	.852									
Q98	.827									
Q99	.829									
Q100	.824									
Q101	.830									
Q102	.820									
Q103						.840				
Q104		.825								
Q105				.832						
Q106		.841								
Q107		.842								
Q108	.839									
Q109		.838								
Q110	.849									

A extraction method: principal component analysis

A rotation method: varimax with Kaiser normalization

**Fig. 1:**
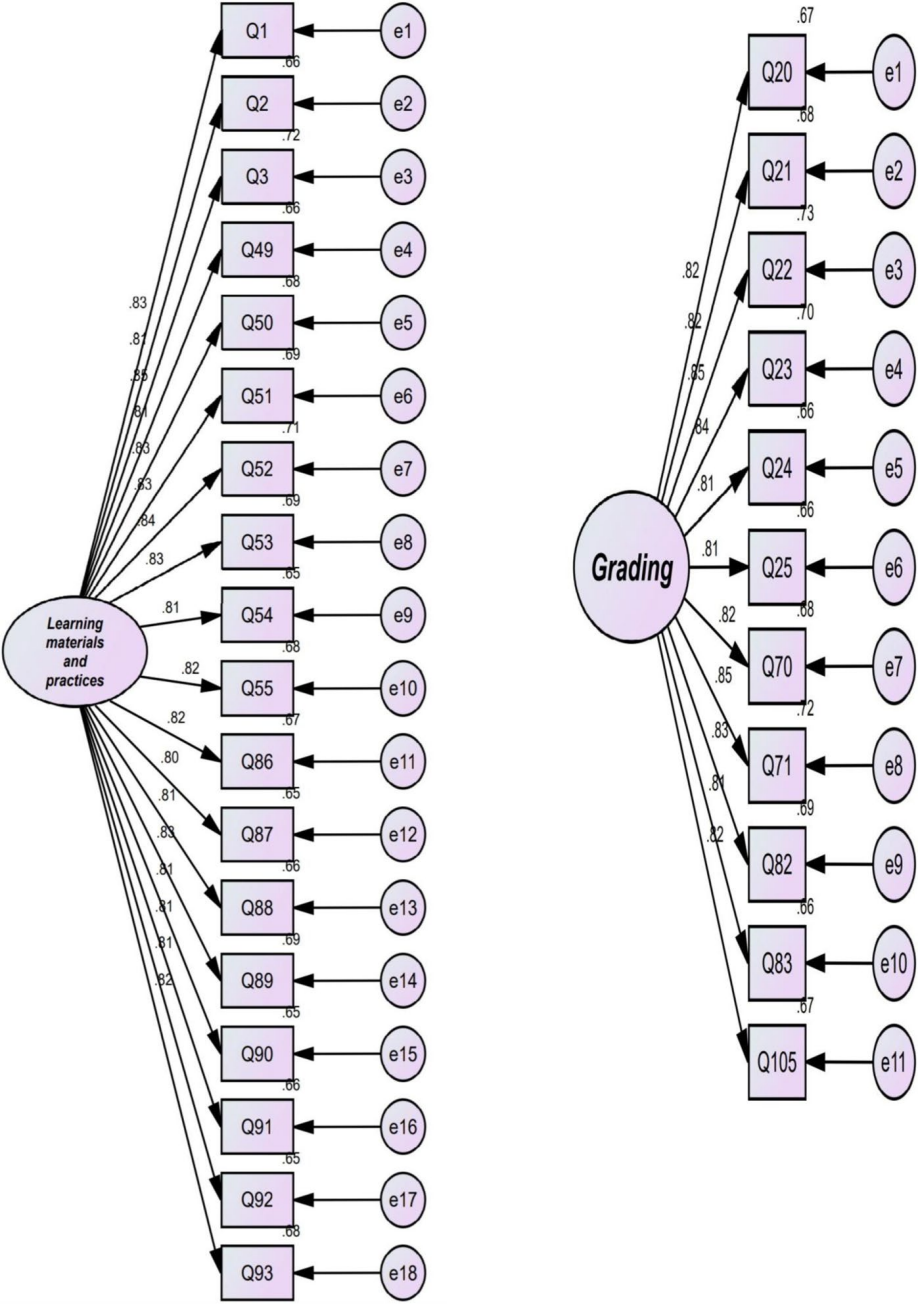

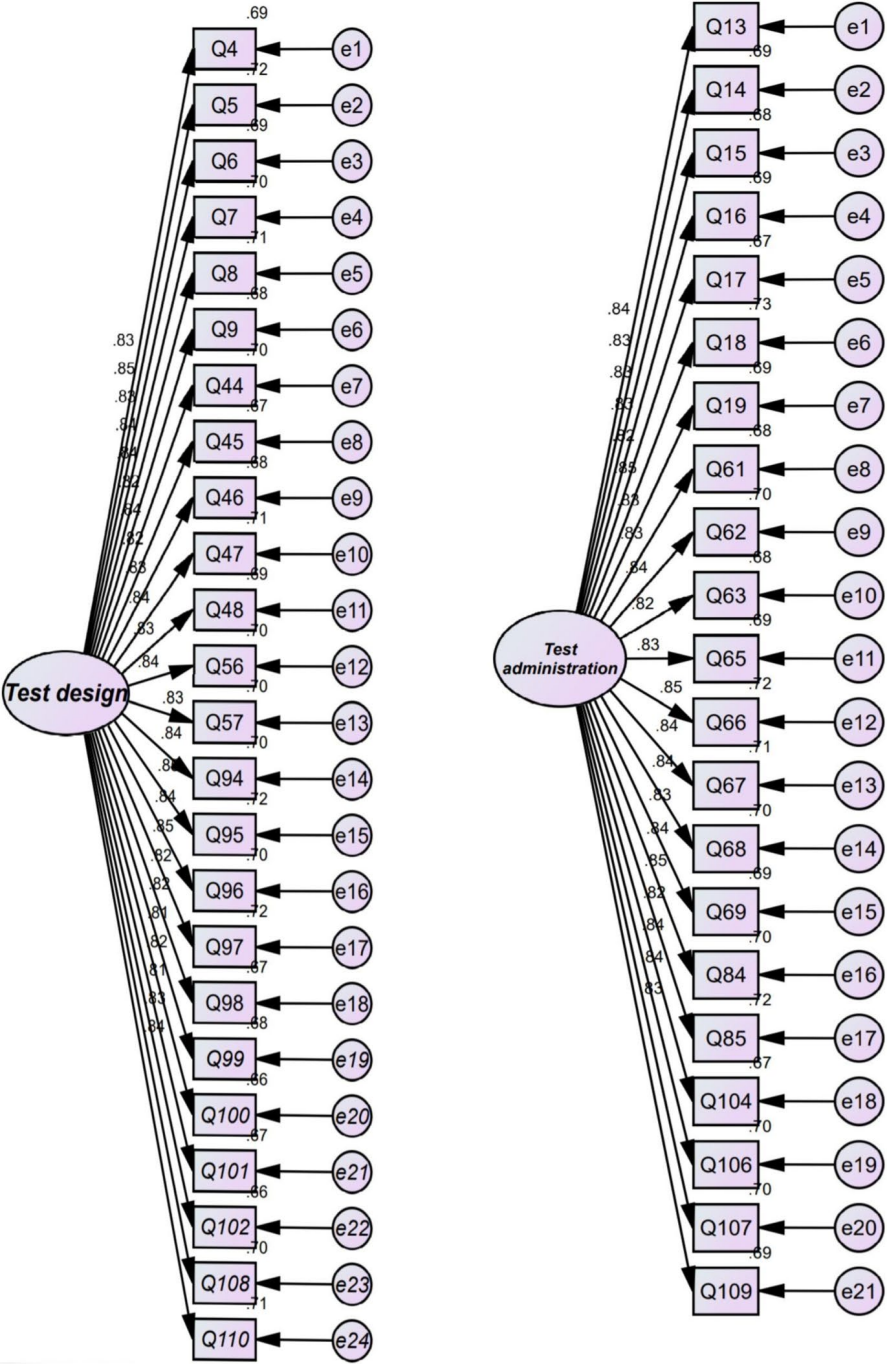

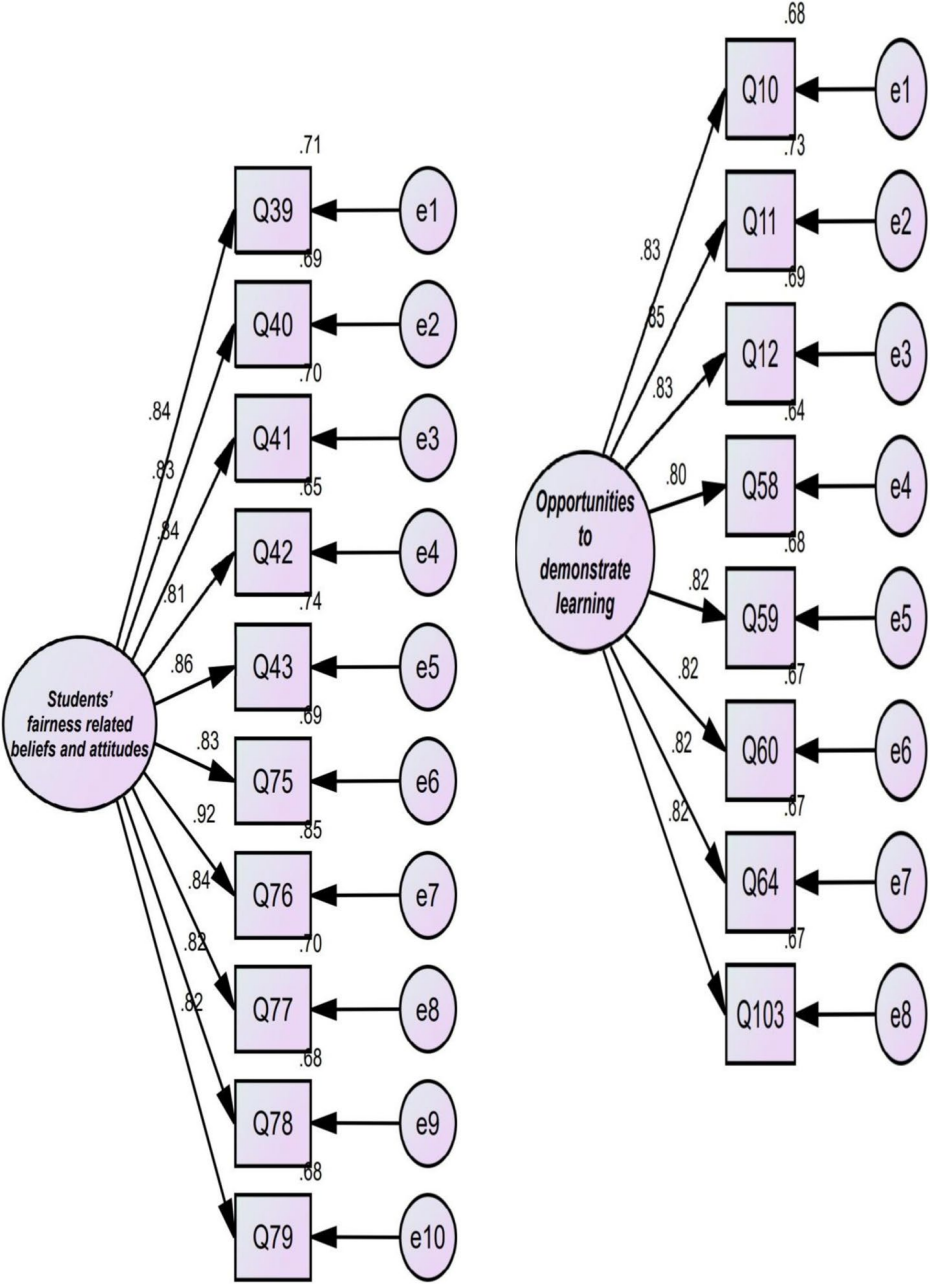

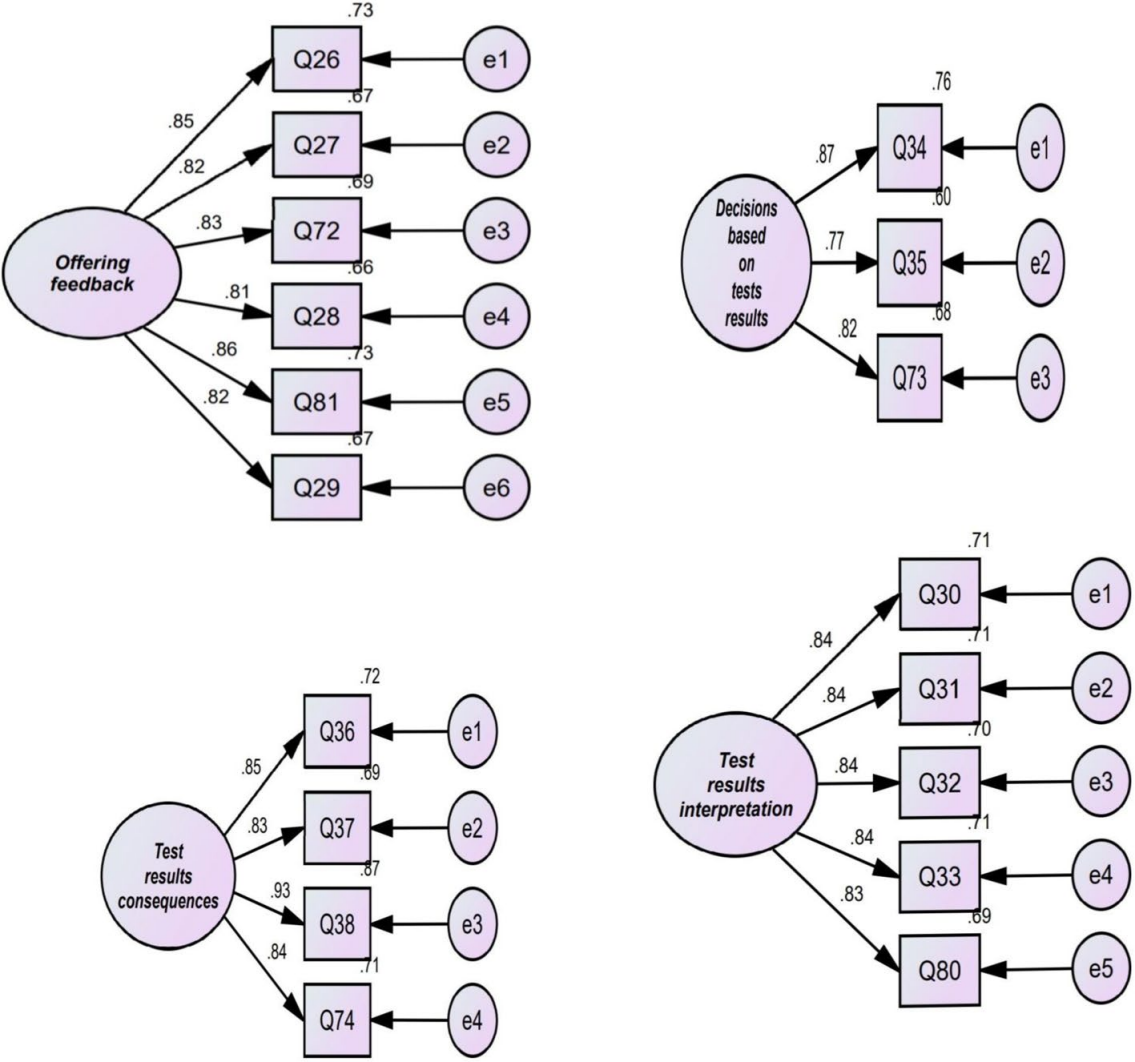
Structural model of the TF showing latent and observed variables and measurement errors

The researcher subjected the 10-factor model which emerged from EFA to CFA using AMOS (see Fig. 1). In the present study, the researcher used *χ*^2^/df (chi-square divided by degree of freedom), goodness of fit index (GFI), root mean square error of approximation (RMSEA), normed fit index (NFI), Tucker and Lewis index (TLI), and comparative fit index (CFI). According to MacCallum et al. (1996), a fit model is acceptable if *χ*^2^/df is less than 3, GFI, NFI, TLI, and CFI are above 0.90, and RMSEA is less than 0.08. As reported in Table 4, the results of CFA showed that all goodness-of-fit indices were above the cutoff points. Hence, the factorial structure of AF was confirmed by CFA.

**Table 4:** Goodness-of-fit indices

Sub-scales	*χ*^2^/df	GFI	NFI	TLI	CFI	RMSEA
Test design	1.24	.93	.96	.99	.99	.02
Test administration	.90	.95	.97	1.00	1.00	.00
Learning materials and practices	.94	.96	.97	1.00	1.00	.00
Grading	1.05	,98	.99	1.00	1.00	.01
Students’ fairness-related beliefs and attitudes	.91	.98	.99	1.00	1.00	.00
Opportunities to demonstrate learning	.80	.98	.99	1.00	1.00	.00
Offering feedback	2.05	.98	.99	.99	.99	.05
Test results interpretation	.60	1.00	1.00	1.00	1.00	.00
Test results consequence	.00	1.00	1.00	1.00	1.00	.00
Decisions based on test results	–	1.00	1.00	–	1.00	.09

The results of the internal consistency of the 10 sub-scales of AFQ are presented in Tables 4 and 5.

**Table 5:** Reliability of the sub-scales and the final version of the questionnaire

Sub-scales	Number of items	Cronbach’s alpha
Learning materials and practices	18	0.974
Test design	24	0.982
Opportunities to demonstrate learning	8	0.944
Test administration	21	0.979
Grading	11	0.959
Offering feedback	6	0.930
Test results interpretation	5	0.923
Decisions based on tests results	3	0.862
Test results consequences	4	0.919
Students’ fairness-related beliefs and attitudes	10	0.960
Overall	110	0.919

As it can be observed, the sub-scales along with the whole questionnaire gained acceptable indexes of Cronbach’s alpha: learning materials and practices (0.97), test design (0.98), opportunities to demonstrate learning (0.94), test administration (0.97), grading (0.95), offering feedback (0.93), tests results interpretation (0.92), decisions based on tests results (0.86), test results consequences (0.91), and students’ fairness-related beliefs and attitudes (0.96). The internal consistency of the whole questionnaire is 0.91 which suggests that AFQ is highly reliable with this sample (Appendix).

## Conclusion

The present study purported to develop and validate a questionnaire to measure fairness in CA within the context of Iranian higher education. Having followed a 12-step systematic procedure, a questionnaire with 10 sub-scales was developed. The construct validity of the questionnaire was supported by CFA and EFA and its reliability was confirmed by Cronbach’s alpha. The hope is that the present questionnaire can be used as a useful instrument to measure the fairness of APs administered in the classroom. This questionnaire is likely to be used for diagnostic purposes by teachers to gauge how much fairness is met in their APs. Additionally, this questionnaire can be used for research purposes to investigate the correlation between fairness in APs and other variables impacting students’ learning. For example, future studies can explore any significant correlation between fairness in APs and students’ motivation to continue learning. Moreover, the 10 sub-scales that emerged from the data are likely to be considered as a new model of fairness in CA in the literature.

Although the present study was an early systematic effort to develop and validate an AFQ, there were two limitations with it that should be acknowledged. The first limitation was related to the sample of the participants. It was limited to two state universities in Iran. Further studies are needed to examine the reliability and validity of the present questionnaire by including other samples of participants (e.g., teachers and school students) in other settings (e.g., schools). The second limitation was germane to the Likert-scale used in the current study. It had only five points (from strongly disagree (1) to strongly agree (5)). By using larger scales, for example with seven to eleven points, researchers are likely to get a better understanding of respondents’ response variance on individual items and, in turn, improve the reliability of the sub-scales. We hope that the implementation of such revisions leads to a more validated questionnaire.

## Data Availability

The datasets used and/or analyzed during the current study are available from the corresponding author on reasonable request.

## Appendix

Table 6

**Table 6:** Test fairness questionnaire

		Strongly disagree	Disagree	Undecided	Agree	Strongly agree
***Learning materials and practices***						
1	I have sufficient access to quality educational resources (e.g., capable teachers and appropriate facilities) to learn the course content.					
2	I have adequate access to test resources (those addressed in tests items) before tests administration.					
3	Given my learning style and abilities, I have enough opportunities to learn in the classroom.					
4	I do not have enough time to prepare for tests.					
5	My teachers do not pay enough attention to my questions and problems in the classroom.					
6	Classes are usually run according to the rules set at the beginning of the semester between the teacher and the students (Everything in the class is based on a schedule and principles agreed upon by all).					
7	I am sufficiently aware of the educational changes (e.g., changes in teaching materials and their presentation) during the semester.					
8	Changes in my learning are not noticed by teachers during the semester (The instruction does not proceed according to my learning conditions).					
9	I do not play an influential role in changing teaching materials during the semester.					
10	My teachers do not have the right to punish me if I do not follow their wishes in the classroom (e.g., to lower my grade or not answer my questions or give me challenging assignments).					
11	I admit that the classroom control is in the hands of my teachers because they are the most knowledgeable and experienced individuals in the classroom (Whatever they say must be done.).					
12	My teachers are capable individuals in the classroom that I love to be known as their students.					
13	My teachers, as a reference in the classroom, have the right to make educational decisions and implement them.					
14	There is a correspondence between the content of the tests items and the materials taught in the classroom.					
15	My teachers do not have adequate pedagogical competence.					
16	My teachers manage the class in a disciplined manner.					
17	I have enough opportunities to work and learn with my classmates in group activities.					
18	My teachers provide a reasonable and adequate explanation of the learning objectives of the semester.					
***Test design***						
1	There is transparency in the design of tests (It is clear who designs them and how).					
2	Assessing my abilities is not affected by my gender (For example, since I look attractive, it does cause my teacher to give me a high grade.).					
3	Tests content does not conflict with my racial/ethnic background (e.g., their content does not contain anything that offends my ethnic customs and values).					
4	Assessing my abilities is not affected by my socioeconomic status (e.g., because I am from the high class of society, it does not cause my teachers to give me a high grade, or because I am from the working class of society, it does not cause my teachers to give me a low grade.).					
5	Tests content does not conflict with my religious beliefs (e.g., their content does not contain materials that challenge or belittle my religious beliefs).					
6	Tests content contradicts my political views and tendencies.					
7	Tests have good design and appearance, proper font, and enough space to answer item questions.					
8	Tests are not designed and tailored to my abilities.					
9	My grades do not change in the tests administered during the semester.					
10	My teachers work together to design tests.					
11	Tests content instills in me a particular ideology (e.g., political or religious).					
12	Tests content does not contradict my cultural values.					
13	There is no fair distribution of power and authority between teachers and me in designing and preparing classroom tests (e.g., everything is under the control of teachers.).					
14	My views and concerns regarding the design and preparation of classroom tests are considered by teachers.					
15	I do not have enough opportunities to assess the abilities of my classmates.					
16	I do not have enough opportunities to assess my abilities in the classroom.					
17	The group’s composition in which I am a member and in which I work has adverse effects on my grade.					
18	My teachers allow me to participate in the design and preparation of classroom tests.					
19	Test items are logically ordered from simple to difficult.					
20	Classroom tests are a combination of close-ended questions (e.g., multiple-choice and fill-in the blank) and open-ended questions (e.g., essay).					
21	Test results can predict my abilities and performance outside of the classroom.					
22	Tests measure exactly what they intend to assess (e.g., grammar tests only assess grammatical competence).					
23	There is an expert group at the university that supervises the preparation and quality of test content.					
***Opportunities to demonstrate learning***						
1	I have enough opportunities to show my abilities.					
2	I do not have enough time to show my abilities during the test administration.					
3	I am familiar with the equipment (e.g., computers), methods (e.g., reading maps), and conditions (e.g., using scheduling time) when taking exams.					
4	There are not enough facilities for students with exceptional physical problems (e.g., blindness, deafness, etc.) to show their abilities.					
5	I cannot afford to pay for tuition and tests expenses.					
6	There are good conditions such as comfortable chairs, a quiet place, optimum light, and suitable temperature during test administrations.					
7	I have easy access to the test site.					
***Test administration***						
1	My teachers provide an adequate explanation on how to answer tests items.					
2	The instructions of tests items are easy to understand.					
3	The conditions of tests administration are the same for all students.					
4	My teachers do not know my name during tests administration.					
5	My teachers answer my questions and interact kindly with me during tests administration.					
6	My teachers treat me kindly during tests administration.					
7	The relationship between my teachers and me is respectful during tests administration.					
8	My teachers provide adequate and clear information during tests administration.					
9	The verbal and non-verbal behaviors of my teachers are respectful to me during tests administration.					
10	My teachers are honest and trustworthy during tests administration.					
11	My teachers allow me to work with them during tests administration.					
12	I cannot freely express my concerns and opinions about how the tests are administered.					
13	My teachers’ interactions with me are influenced by my gender during test administration (For example, because I have an attractive appearance, my teachers pay more attention to me).					
14	My teachers’ interactions with me are not affected by my race/ethnicity (For example, because teachers and I are of the same ethnicity, it does not make them interacts with me differently).					
15	My teachers’ interactions with me are not affected by my socioeconomic status (social class) during tests administration (For example, because I come from a wealthy family, it does not cause my teachers to interact with differently.).					
16	My teachers’ interactions with me are not influenced by my religious beliefs during tests administration (For example, because my religion is different from that of my teachers, this does not mean that they interact with me differently).					
17	My teachers’ interactions with me are not influenced by my political views during test administration (For example, because my teachers know that I do not have political views close to him, they interact less with me).					
18	My teachers’ interactions with me are not affected by our friendship and relationship during tests administration (For example, because I have a close relationship with a teacher, this does not mean that he/she interacts with me differently).					
19	There is no possibility of cheating during tests administration.					
20	It is possible to rest, eat or go to WC during tests administration.					
21	There is no a group of experts at the university to supervise how tests are administered.					
***Grading***						
1	There is no consistency in grading criteria (My teachers change scoring criteria continuously).					
2	My teachers provide a reasonable and adequate explanation of grading criteria.					
3	There is transparency in announcing test results (It is clear when, how and by whom test results are announced.).					
4	There is no consistency in announcing test results (It is clear when, how and by whom test results are announced.).					
5	My grades are kept confidential in the classroom.					
6	My teachers do not provide sufficient and valid information about grading criteria at the beginning of the semester.					
7	My teachers announce test results and grades on time.					
8	My teachers allow me to cooperate in determining grading criteria.					
9	My new grades are not affected by my previous grades.					
10	When I get a low grade, there is not enough opportunity to make up for it.					
11	Scoring my abilities is not affected by my friendship with teachers (For example, if I have a close relationship with a teacher, this does not mean that he/she gives me a score higher than my abilities).					
12	There is an expert group at my university supervising how tests are scored.					
***Offering feedback***						
1	The feedback that teachers offer on my performance is not clear to me.					
2	The feedback that teachers offer on my performance is in line with the educational materials taught during the semester.					
3	The feedback that teachers offer on my performance is reasonable and adequate.					
4	The rewards given by teachers regarding test results are equal for all students.					
5	The punishments imposed by teachers regarding test results are equal for all students (For example, if a student does not obtain an intended grade, he/she should take some compensatory actions).					
6	My teachers listen carefully to my requests for a review of my grade.					
***Tests results interpretation***						
1	My grades are not interpreted clearly (The criteria to interpret my grades are not specified).					
2	My grades are interpreted consistently (For example, if my grade is lower than ten, it always means that I have to retake the course).					
3	Teachers express their reasons for my grades interpretations.					
4	Test results effects are equal for all students (For example, if the passing score is ten, all students whose score is lower than ten will not pass the course).					
5	There is no fair balance between the time and effort I put into preparing for tests and the results I get.					
***Decisions based on tests results***						
1	The decisions made based on my grades are clear to me (It is clear what a decision is made for each grade by the teacher).					
2	The decisions made based on my grades are consistent over the semester (For example, whenever I get a low grade, my teachers do not change it).					
3	The decisions made based on my grades are not logical and justifiable for me (For example, if my grade is lower than ten, I will not pass that course).					
4	Decisions made based on my grades are announced in a timely manner.					
***Test results consequences***						
1	Tests have adverse effects on my learning.					
2	Tests have adverse effects on my society (For example, tests make everything be summed up in getting a degree).					
3	Tests have adverse effects on my family (For example, when I am going to take a test, my all family members have to quit their affairs and help me).					
4	Tests results affect my future career.					
***Students’ fairness-related beliefs and attitudes***						
1	What has happened in my life has been fair.					
2	The world where I live in is an unjust place.					
3	I have control over my life events.					
4	I have positive attitudes toward university and its education system.					
5	I have negative attitudes toward my teachers.					
6	I have negative attitudes toward tests.					
7	I feel confident when I take a test.					
8	I attribute test results to my inner abilities.					
9	All individuals have equal opportunities to study in my country.					

## Abbreviations

CA: Classroom assessment
APs: Assessment practices
